# *Parasogata* gen. n., a new genus of the tribe Delphacini with descriptions of two new species from China (Hemiptera, Fulgoromorpha, Delphacidae)

**DOI:** 10.3897/zookeys.806.26394

**Published:** 2018-12-13

**Authors:** Zheng-Xiang Zhou, Lin Yang, Xiang-Sheng Chen

**Affiliations:** 1 Institute of Entomology, Guizhou University, Guiyang, Guizhou, 550025, P.R. China Guizhou University Guizhou China; 2 College of Agriculture, Anshun University, Anshun, 561000, P.R. China Anshun University Anshun China; 3 Guizhou Key Laboratory for Plant Pest Management of Mountainous Region, Guizhou University, Guiyang, Guizhou, 550025, P.R. China Guizhou University Guizhou China; 4 Special Key Laboratory for Development and Utilization of Insect Resources of Guizhou, Guizhou University, Guiyang, Guizhou, 550025, P.R. China Anshun University Anshun China

**Keywords:** Delphacid, distribution, Fulgoroidea, new taxa, planthopper

## Abstract

A new planthopper genus *Parasogata***gen. n.** (Delphacidae: Delphacinae: Delphacini) was described and illustrated with two new species *P.binaria***sp. n.** and *P.furca***sp. n.** from south China. A key to species of the new genus is also given.

## Introduction

The planthopper tribe Delphacini Leach, 1815 is the largest clade of Delphacidae, occurring in all ecoregions (excluding Antarctica) and including approximately 1652 species in 319 genera (Bourgoin 2018). In China, 259 species in 135 genera are known (Ding 2006; Dong and Qin 2012; Hou et al. 2013, 2014a, b; Qin 2005, 2006, 2007; Qin et al. 2006, 2008, 2009a, b, 2010, 2011, 2012, 2014; Ren et al. 2015).

Here, a new genus, *Parasogata* gen. n., with two new species, *P.binaria* sp. n. and *P.furca* sp. n., are described and illustrated from China. The new genus is assigned to the Delphacini because the spinal formula of the hind leg 5–7–4, tibial spur large, thin, flattened and bearing a row of fine, black-tipped teeth on the posterior margin; genital diaphragm developed, suspensorium present. The similarities and affinities of the new genus with similar genera are compared and discussed. A key to the species of the new genus is also provided.

## Materials and methods

Terminology of morphological and measurements follow Yang and Yang (1986) and the morphological terminology of female genitalia follows Bourgoin (1993). Measurements of body length equal the distance between the apex of vertex and tip of tegmen. All measurements are in millimeters (mm). Dry specimens were used for the description and illustration. Color pictures for adult habitus were obtained by KEYENCE VHX-1000. External morphology was observed under a stereoscopic microscope Leica Mz 12.5 and characters were measured with an ocular micrometer. The genital segments of the examined specimens were macerated in 10% KOH and drawn from preparations in glycerin jelly using Olympus CX41 and Leica MZ 12.5 stereomicroscope. Illustrations were scanned with Canon CanoScan LiDE 200 and imported into Adobe Photoshop 6.0 for labeling and plate composition.

The type specimens of the new species are deposited in the Institute of Entomology, Guizhou University, Guiyang, Guizhou Province, China (**GUGC**).

## Taxonomy

### Genus Parasogata gen. n.

Taxon classificationAnimaliaHemipteraDelphacidae

http://zoobank.org/C168D41B-DB7F-4841-BCCC-EF3299BD2EC6

Figs 9–16 , 17–29 , 30–34 , 35–42 , 43–55 , 56–60

#### Type species.

*Parasogatabinaria* sp. n.

#### Diagnosis.

This genus is readily recognized by its large size and vertex, pronotum and mesonotum bearing an uninterrupted white fascia. The genus is most similar to *Sogata* Distant, 1906 but separately by the phallus being up-curved (down-curved in *Sogata* (Ding 2006: figs 281–283)), with a row processes at subapically (without process in *Sogata*).

#### Description.

General color of male yellowish white to brown (Figs 9–12, 35–38). Vertex, pronotum and mesonotum with an uninterrupted white fascia (Figs 9, 35). Vertex, frons, face, antennae yellowish brown to yellowish white (Figs 9–12, 35–38). Pronotum and mesonotum yellowish white (Figs 13, 39). Forewings and hindwings hyaline (Figs 9–12, 35–38). Legs yellowish white (Figs 10, 36). Abdomen yellow (Figs 10, 36). Head including eyes narrower than pronotum (Figs 13, 15, 39, 41). Vertex subquadrate, anterior margin arched, lateral carinae with slightly concave, submedian carinae uniting at apex. Frons with single median carina, longer in middle line than wide at widest part, widest at apex (Figs 14, 16, 40, 42). Y-shaped carina feeble (Figs 13, 15, 39, 41). Antennae cylindrical, with basal segment shorter than second, reaching frontoclypeal suture (Figs 14, 16, 40, 42). Pronotum with lateral carinae almost attaining hind margin (Figs 13, 15, 39, 41). Posttibial spur with 29–32 distinct teeth along hind margin.

#### Male genitalia.

Anal segment collar-shaped, lateroapical angles produced into processes (Figs 24–25, 50–51). Pygofer in profile wider ventrally than dorsally, laterodorsal angles not produced, in posterior view with opening wider than long, lateral margins well defined, lateral quadrate areas strongly sclerotized, medioventral process absent (Figs 21–23, 47–49). Diaphragm broad (Figs 23, 49). Aedeagus long, tubular, with a row processes at subapically, slightly upward apically (Figs 26, 52). Genital styles simple, widely divergent apically (Figs 27–28, 53–54). Suspensorium large (Figs 29, 55).

#### Etymology.

This generic name “*Parasogata*” refers to its strong similarity to *Sogata*. The name is to be treated as feminine.

#### Distribution.

China.

#### Remarks.

**Figures 1–8.**
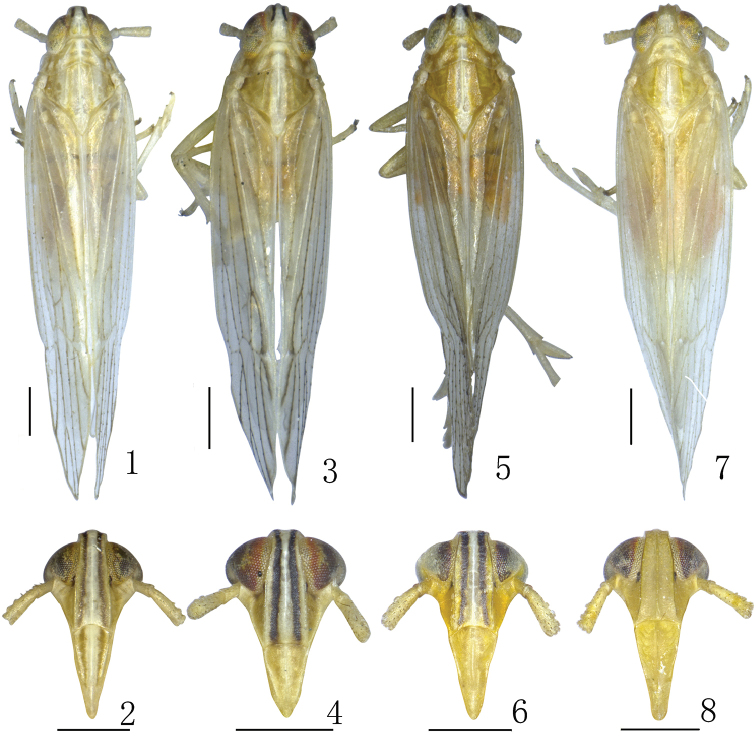
Dorsal and frontal view **1, 2***Neometopinapenghuensis* Yang, 1989 **3, 4***Sogatadohertyi* Distant, 1906 **5, 6***Neunkanodesformosana* Yang, 1989 **7, 8***Lisogatazhejiangensis* Ding, 2006. Scale bar: 0.5 mm.

The genus *Parasogata* gen. n. resembles *Sogata* Distant, 1906, *Neometopina* Yang, 1989, *Neunkanodes* Yang, 1989 and *Lisogata* Ding, 2006 in vertex, pronotum and mesonotum with an uninterrupted white fascia, frons with median carina single (Figs 1–8), but differs from these genera by anal segment with two pairs of processes, or with a pair of forked processes (without process in *Lisogata*); aedeagus not forked at half of apex (with forked at half of apex in *Neometopina* and *Neunkanodes*); aedeagus with processes and decurved dorsad apically (without process and decurved ventrad apically in *Sogata*) (Table 1).

**Table 1.** Differences among *Parasogata* and similar genera.

	*Parasogata* gen. n.	* Neometopina *	* Sogata *	* Neunkanodes *	* Lisogata *
Size (mm)	4.72–5.20	4.62–4.82	4.20–4.70	4.40–4.90	4.40–4.70
Frons color	Black with median carina yellowish white	Brown with median carina yellowish white	Black with median carina yellowish white	Brown with median carina yellowish white	Yellow
Y-shaped carina	Feeble	Distinct	Feeble	Feeble	Feeble
Lateral carinae of pronotum	Almost attaining hind margin	Almost attaining hind margin	Almost attaining hind margin	Conspicuous not attaining hind margin	Almost attaining hind margin
Number of teeth of hind tibial spur	29–32	20–23	18–23	23–24	30–38
Hind margin of male pygofer	Not produced	Not produced	Not produced	Produced caudad, lobe-like	Not produced
Processes of male anal segment	Two pairs or one pair with bifurcation	One pair	One pair	One pair	None
Apex of aedeagus	Unforked	Forked	Unforked	Forked	Unforked
Inner basal angle of genital styles	None	None	None	Protruding	None

##### Revised couplets to the key to Chinese Delphacini by Ding (2006)

**Table:** 

70	Pygofer in profile with posterior margin produced caudad in a lobe (Ding 2006: fig. 314)	***Neunkanodes* Yang**
–	Pygofer in profile not produced	**71a**
71a	Aedeagus without processes, apically decurved ventrally (Ding 2006: figs 281–283)	***Sogata* Distant, 1906**
–	Aedeagus with distinct processes, apically recurved dorsally (Figs 26, 52)	***Parasogata* gen. n.**

##### Key to species of genus *Parasogata* gen. n. (male)

**Table:** 

1	Pronotum brown except median carina yellowish white (Figs 13, 17); anal segment with two pairs of processes, each with apex not forked (Figs 24–25)	***P.binaria* sp. n.**
–	Pronotum yellow except median carina yellowish white (Figs 39, 43); anal segment with a pair of processes, each with apex forked (Figs 50–51)	***P.furca* sp. n.**

### Parasogata binaria sp. n.

Taxon classificationAnimaliaHemipteraDelphacidae

http://zoobank.org/ACC2E082-45A0-4653-9B55-52B8E0182662

Figs 9–16 , 17–29 , 30–34

#### Type material.

Holotype: ♂, CHINA, Yunnan: Daweishan National Natural Reserve (22°81'N, 103°79'E), 18 Aug. 2017, Y.-J. Sui. Paratypes: 1♂1♀, same data as holotype; 1♂2♀♀, same data as holotype except, 22 Aug. 2017, Q. Luo; 6♂♂5♀♀, same data as holotype except, 19 Aug. 2017, N. Gong.

#### Measurements.

Body length (from apex of vertex to apex of forewing): male 5.10–5.20 mm (n = 9); female 5.90–6.00 mm (n = 8); forewing length: male 4.30–4.42 mm (n = 9); female 5.10–5.12 mm (n = 8).

#### Diagnosis.

Big-sized species with general color yellowish white to yellowish brown, anal segment with two pairs of spinose processes; aedeagus with ten processes subapically and with irregular teeth on ventral side of apex, constriction and bluntly rounded at apex (Fig. 26).

#### Description.

**Figures 9–16.**
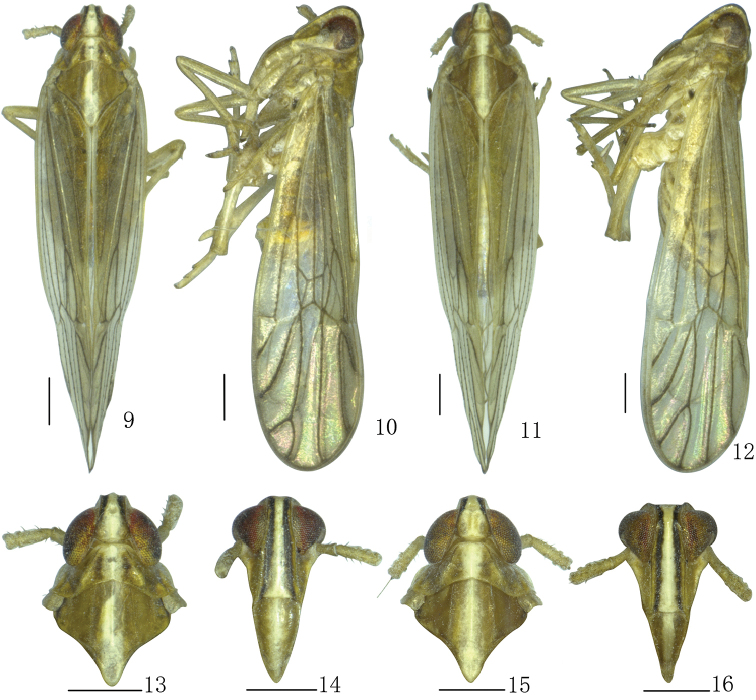
*Parasogatabinaria* sp. n. **9, 10** Male habitus (dorsal and lateral views) **11, 12** Female habitus (dorsal and lateral views) **13** Male head and thorax, dorsal view **14** Male front **15** Female head and thorax, dorsal view **16** Female front. Scale bar: 0.5 mm.

*Coloration.* General color yellowish white to yellowish brown. Head yellowish brown (Figs 13–16). Vertex yellowish white, except along lateral margin dark brown (Figs 13–16). Frons black, except median carinae yellowish white and lateral margins yellowish brown (Fig. 14). Clypeus and genae yellowish brown (Fig. 14). Rostrum yellowish brown, with apex brown. Eyes generally yellow to brown (Figs 9–16), ocelli dark brown (Figs 10, 12, 14, 16). Antennae yellow (Figs 13–16). Pronotum and mesonotum yellowish brown, except media carinae yellowish white (Figs 13, 15). Forewings with veins dark brown (Figs 9–12). Hindwings pale white, with veins brown. Legs yellowish white to pale yellow, tibiae pale yellow basally, tarsomeres yellowish white (Figs 10, 12). Abdomen brown, except lateral margins yellow (Figs 9–12).

**Figures 17–29.**
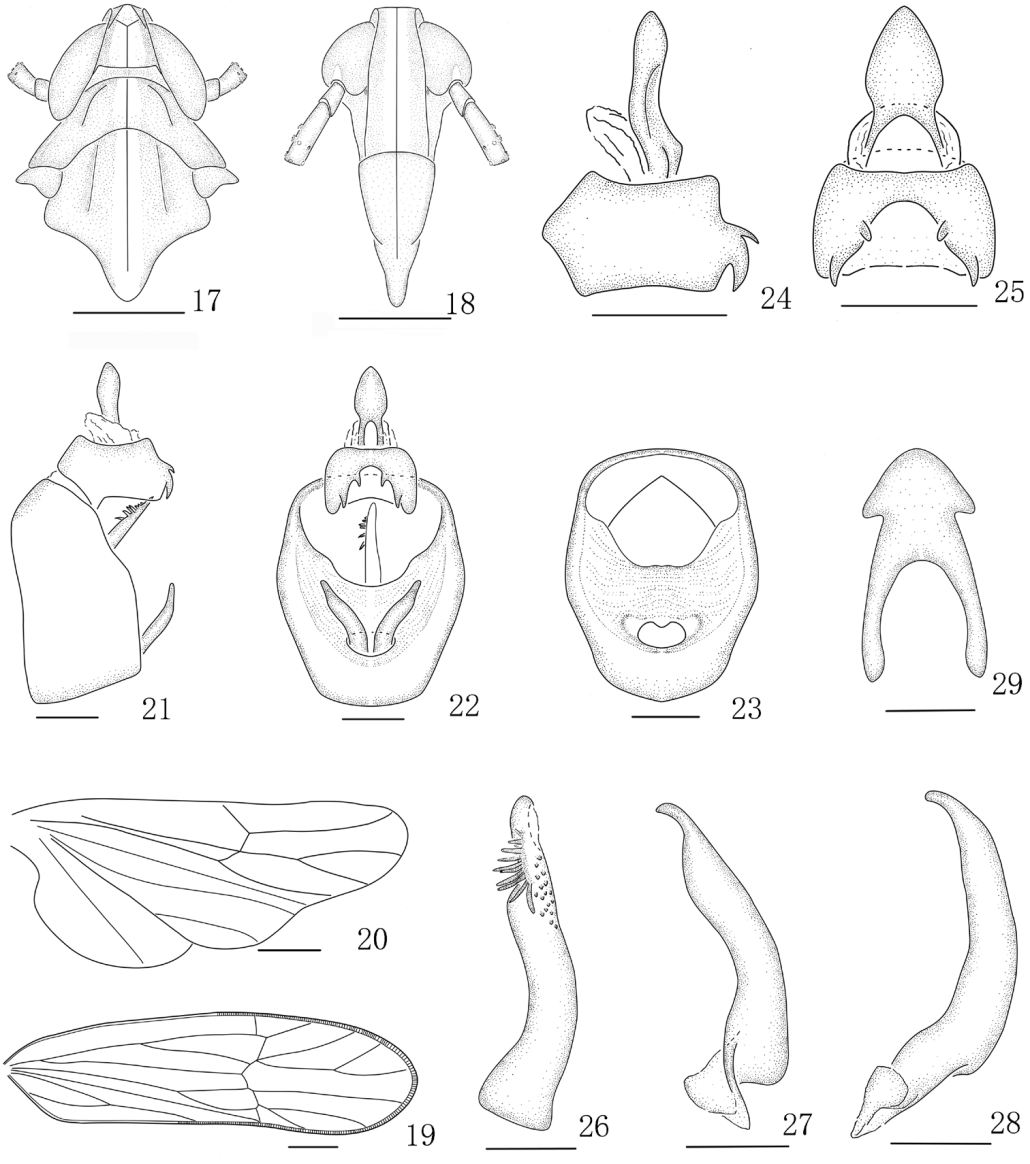
*Parasogatabinaria* sp. n. male **17** Head and thorax, dorsal view **18** Front **19** Forewing **20** Hindwing **21** Genitalia, lateral view **22** Genitalia, caudal view **23** Diaphragm, caudal view **24** Anal segment, left view **25** Anal segment, caudal view **26** Aedeagus, left view **27** Genital style, caudal view **28** Genital style, left view **29** Suspensorium. Scale bars: 0.5 mm (**17–23**); 0.2 mm (**24–25**); 0.1 mm (**26–29**).

*Structure.* Head including eyes narrower than pronotum, ratio 0.77:1 (Figs 13, 15). Vertex with anterior margin arched, lateral carinae slightly concave, submedian carinae uniting at apex, longer than wide at base, ratio 1.28:1, narrower at apex than at base, ratio 0.64:1 (Figs 13, 17). Frons longer in middle line than wide at widest part, ratio 2.28:1 (Figs 14, 18), lateral carinae nearly straight (Figs 14, 18). Postclypeus wider at base than frons at apex, slightly longer than wide at base (Figs 14, 18). Antennae cylindrical, basal segment longer than wide, ratio 1.55:1, shorter than second, ratio 0.42:1 (Figs 14, 18). Pronotum shoter than vertex, ratio 0.75:1 (Figs 13, 17). Mesonotum longer than pronotum and vertex combined, ratio 1.25:1 (Figs 13, 17). Posttibial spur with approximately 30–32 distinct teeth along hind margin. Forewings longer than widest part, ratio 3.48:1, widest at apical 1/4 (Figs 10, 12, 19).

*Male genitalia.* Anal segment with two pairs of spinose processes, upper pair smaller (Figs 24–25). Pygofer quadrate in posterior view (Figs 21–23). Diaphragm broad, transparent (Fig. 23). Aedeagus with ten processes subapically and with irregularity teeth at ventral of apex, constriction and blunt rounded at apex (Fig. 26). Genital styles with inner margin arched and outer margin concave in caudal view, distinctly constricted at apex (Figs 27–28). Suspensorium large and arrow-shaped (Fig. 29).

*Female genitalia.* Gonocoxa VIII at base of inner margin arched (Fig. 31). Gonapophyses VIII with apex sharp, with ventral margin membranous at half of apex, dorsal aspect with several small teeth apically (Fig. 32). Gonapophyses IX long, sclerotized, curved basally, narrowing towards apex, with approximately 17 teeth, abruptly reduced and indistinct at apex (Fig. 33). Gonoplacs twisted, long and stripe-shaped (Fig. 34).

**Figures 30–34.**
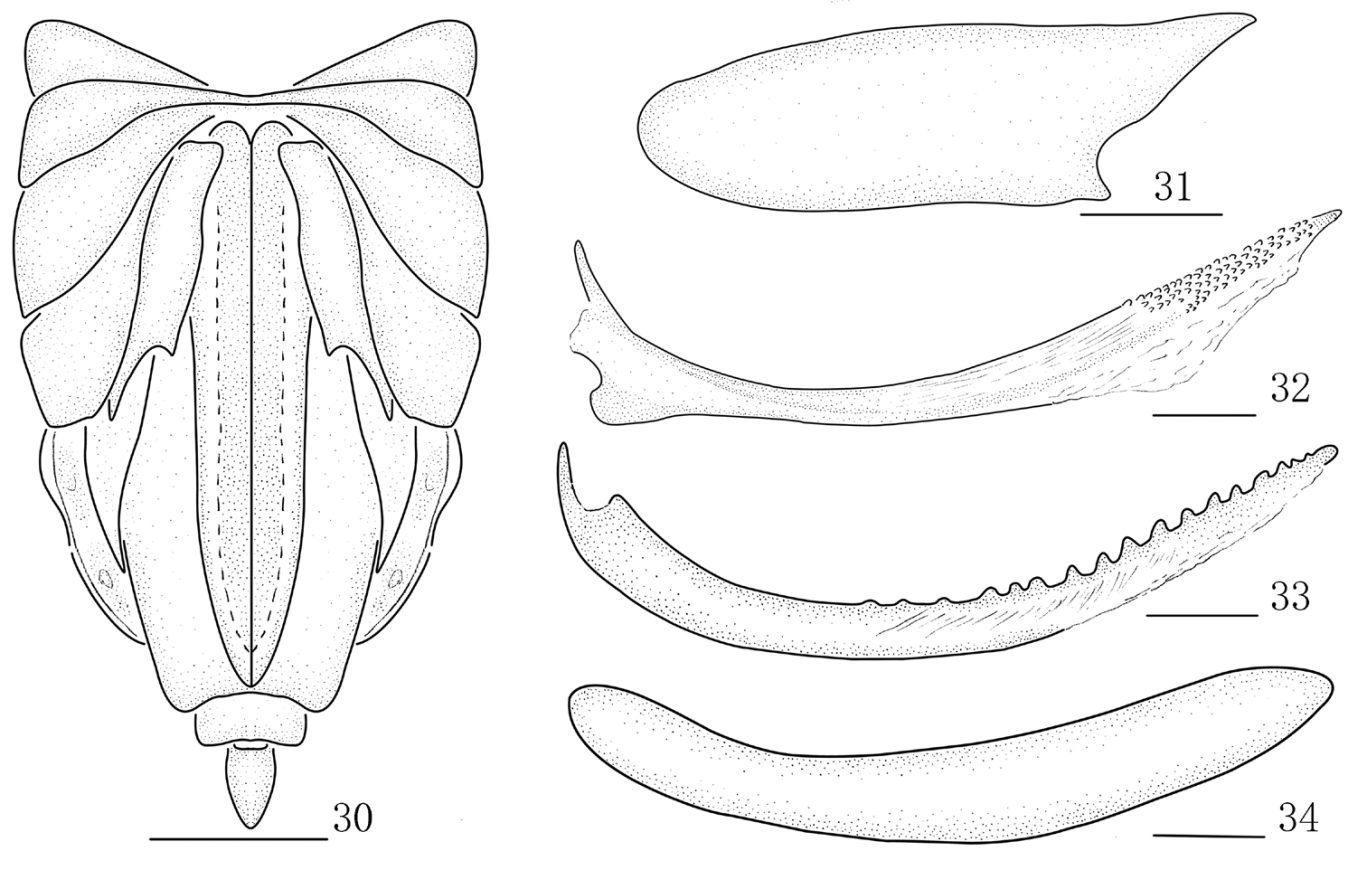
*Parasogatabinaria* sp. n., female **30** Abdomen, ventral view **31** Gonocoxa VIII **32** Gonapophysis VIII **33** Gonapophysis IX **34** Gonoplac. Scale bars: 0.5 mm (**30**); 0.2 mm (**31–34**).

#### Report hosts.

None.

#### Distribution.

China (Yunnan).

#### Etymology.

The specific epithet is from the Latin word *binaria* (bipartite), referring to the anal segment with two pairs of processes.

#### Remarks.

The species is similar to *Sogatadohertyi* (Distent, 1906), but can be distinguished by anal segment with processes cross (not cross in *Sogatadohertyi*), aedeagus with a row processes (without process in *Sogatadohertyi*).

### Parasogata furca sp. n.

Taxon classificationAnimaliaHemipteraDelphacidae

http://zoobank.org/85CEB0C9-63CD-42F2-B312-9AD19FAD4F40

Figs 35–42 , 43–55 , 56–60

#### Type material.

Holotype: ♂, **CHINA**, **Guizhou**: Wangmo County, Zhexiang (24°97'N, 106°15'E), 7 Jul. 2016, H.-X. Li and L.-J. Yang. Paratypes: 3♂♂, same data as holotype: 2♂♂1♀, **Yunnan**: Yuanjiang County, Dongezhen (23°69'N, 101°82'E), 26 Aug. 2014, Z.-X. Zhou.

#### Measurements.

Body length (from apex of vertex to apex of forewing): male 4.72–4.84 mm (n = 6), female 5.10–5.22 mm (n = 1); forewing length: male 3.61–3.93 mm (n = 6); female 4.42–4.51 mm (n = 1).

#### Diagnosis.

Big-sized species with General color yellow, anal segment with a pair of spinose processes, forked apically (Figs 50–51); aedeagus with eight processes and with many irregularity ventral teeth at subapically (Fig. 52).

#### Description.

**Figures 35–42.**
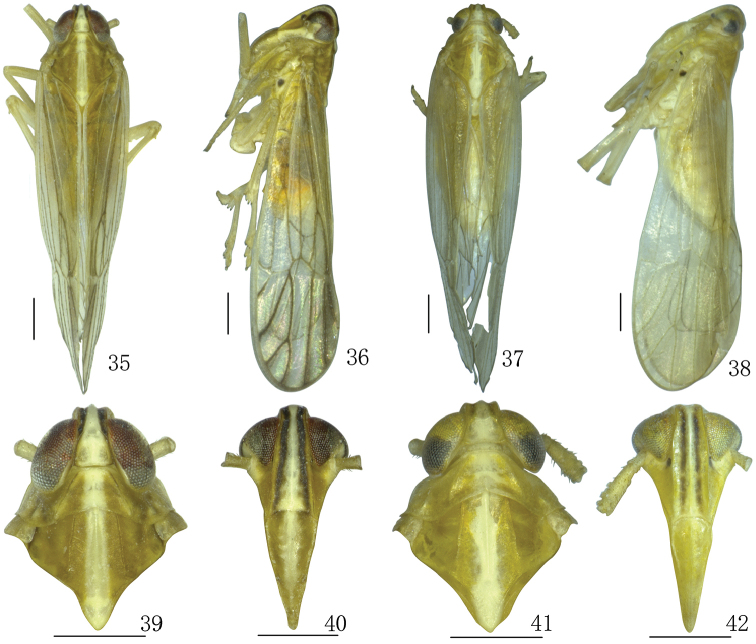
*Parasogatafurca* sp. n. **35, 36** Male habitus (dorsal and lateral views) **37, 38** Female habitus (dorsal and lateral views) **39** Male head and thorax, dorsal view **40** Male front **41** Female head and thorax, dorsal view **42** Female front. Scale bar: 0.5 mm.

*Coloration.* Head yellow. Vertex yellowish white to black (Figs 39, 41). Frons black except middle carinae yellowish white and lateral margin yellowish brown (Figs 40, 42). Clypeus and genae yellow (Figs 40, 42). Rostrum yellowish brown, with apex brown. Eyes generally yellow to brown (Figs 35–42), ocelli yellowish brown (Figs 36, 38, 40, 42). Antennae yellow (Figs 35–42). Pronotum and mesonotum with carinae yellowish brown (Figs 39, 41). Forewings with veins dark brown (Figs 35–38). Hindwings pale white, veins brown. Legs yellowish white to pale yellowish; tibiae yellow basally, tarsomeres yellowish white (Figs 36, 38). Abdomen yellow (Figs 35–38).

**Figures 43–55.**
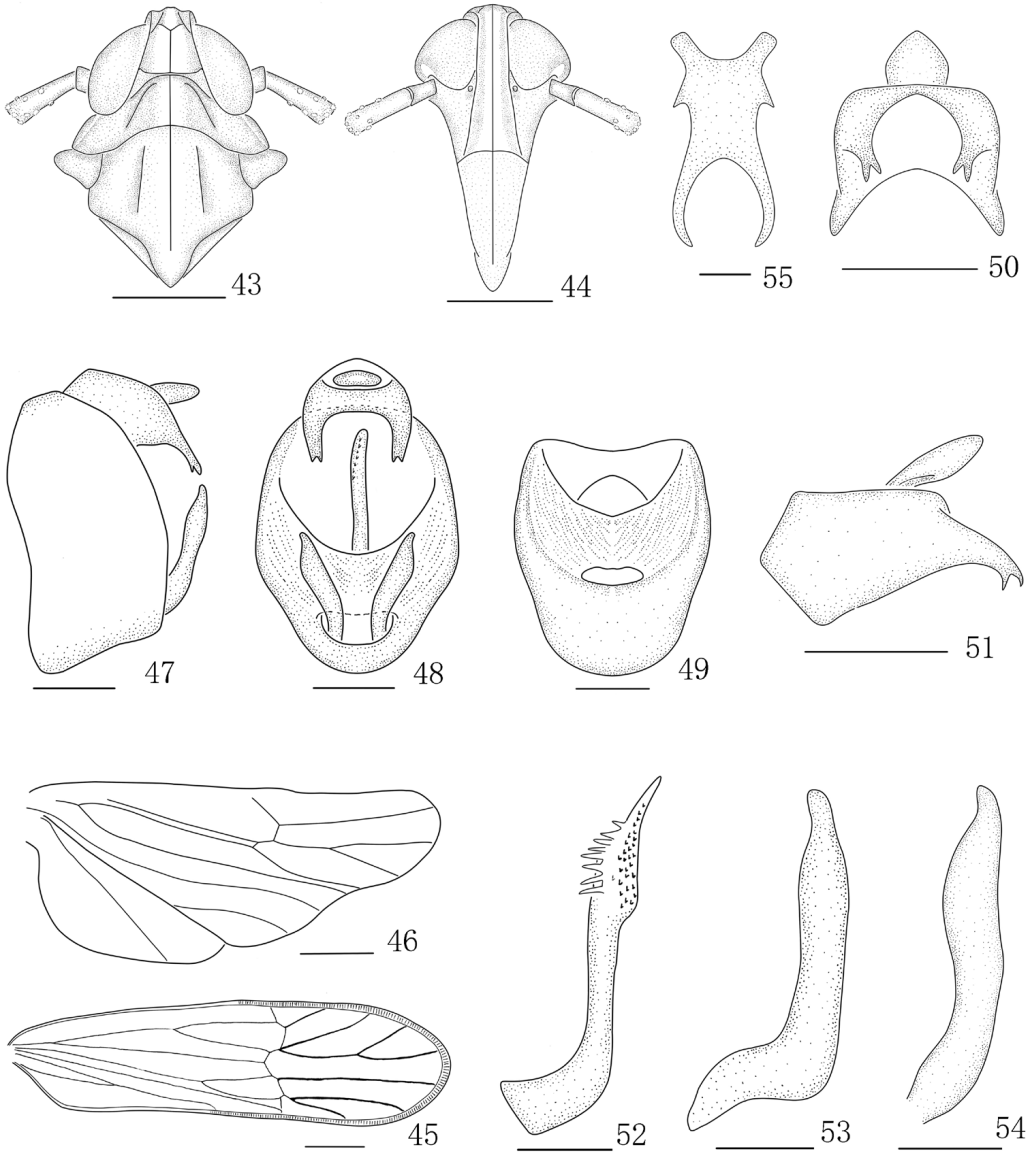
*Parasogatafurca* sp. n., male **43** Head and thorax, dorsal view **44** Front **45** Forewing **46** Hindwing **47** Genitalia, lateral view **48** Genitalia, caudal view **49** Diaphragm, caudal view **50** Anal segment, caudal view **51** Anal segment, left view **52** Aedeagus, left view **53** Genital style, left view **54** Genital style, caudal view **55** Suspensorium. Scale bars: 0.5 mm (**43–49**); 0.2 mm (**50, 51**); 0.1 mm (**52–55**).

*Structure.* Head including eyes narrower than pronotum, ratio 0.79:1 (Figs 39, 43). Vertex with anterior margin transverse, lateral carinae with slightly concave, longer than wide at base, ratio 1.22:1, narrower at apex than at base, ratio 0.55:1 (Figs 39, 43). Frons longer in middle line than wide at widest part, ratio 1.83:1, lateral carinae straight (Figs 40, 44). Postclypeus wider at base than frons at apex, slightly longer than wide at base (Figs 41, 46). Antennae cylindrical, basal segment longer than wide, ratio 1.55:1, shorter than second, ratio 0.38:1 (Figs 39–44). Pronotum shorter than vertex, ratio 0.67:1 (Figs 36, 38, 40, 42, 44). Mesonotum longer pronotum and vertex combined, ratio 1.21:1 (Figs 39, 43). Forewings longer than widest part, ratio 3.85:1, widest at apical 1/4 (Figs 35–38, 45). Posttibial spur with approximately 29–32 distinct teeth along hind margin.

*Male genitalia.* Anal segment with a pair of spinose processes, forked apically (Figs 50–51). Pygofer quadrate in caudal view (Figs 47–49). Diaphragm broad, transparent, dorsal margin arched (Fig. 49). Aedeagus with eight processes and with many irregularity ventral teeth at subapically (Fig. 52). Genital styles with lateral margins arched in caudal view, with two lateral margins almost parallel in profile (Figs 53–54). Suspensorium large and X-shaped, with a process at each lateral margin (Fig. 55).

*Female genitalia.* Gonocoxa VIII with base of inner margin slightly concave (Fig. 57). Gonapophyses VIII with apex sharp, ventral margin membranous at half of apical, in dorsal margins with several small teeth at half of apical (Fig. 58). Gonapophyses IX long, sclerotized, curved basally and narrowing towards apex, serrated caudad in distal, with approximately 18 teeth, abruptly reduced and indistinct at apex (Fig. 59). Gonoplacs twisted (Fig. 60).

**Figures 56–60.**
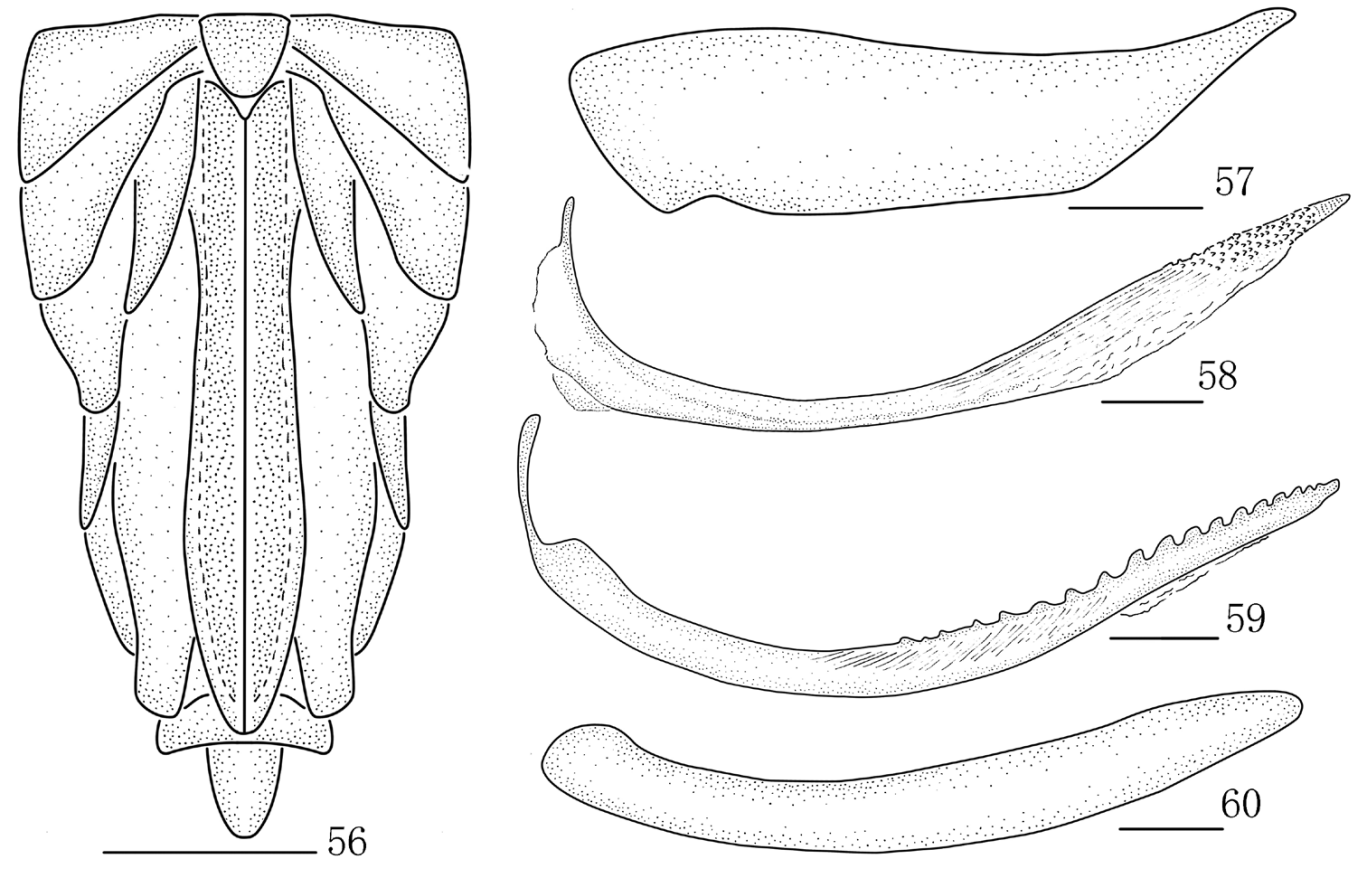
*Parasogatafurca***sp. n.**, female **56** Abdomen, ventral view **57** Gonocoxa VIII **58** Gonapophysis VIII **59** Gonapophysis IX **60** Gonoplac. Scale bars: 0.5 mm (**56**); 0.2 mm (**57–60)**.

#### Report hosts.

None.

#### Distribution.

China (Guizhou, Yunnan).

#### Etymology.

The specific epithet is from the Latin word *furca* (forked), indicating the anal segment produced lateroapical angles forked.

#### Remarks.

This species is similar to *Parasogatabinaria* sp. n., but can be distinguished by the anal segment with a single pair of processes (two pairs of processes in *Parasogatabinaria* sp. n.), suspensorium with dorsal margin hunch-up (with dorsal margin concave in *Parasogatabinaria* sp. n.).

## Supplementary Material

XML Treatment for Parasogata

XML Treatment for Parasogata binaria

XML Treatment for Parasogata furca
